# Semi-Parallel logistic regression for GWAS on encrypted data

**DOI:** 10.1186/s12920-020-0724-z

**Published:** 2020-07-21

**Authors:** Miran Kim, Yongsoo Song, Baiyu Li, Daniele Micciancio

**Affiliations:** 1 School of Biomedical Informatics, University of Texas, Health Science Center at Houston, Houston, 77030 TX USA; 2 Microsoft Research, Redmond, 98052 WA USA; 3 Department of Computer Science and Engineering, University of California-San Diego, San Diego, 92093 CA USA

**Keywords:** Homomorphic encryption, Genome-wide association studies, Logistic regression

## Abstract

**Background:**

The sharing of biomedical data is crucial to enable scientific discoveries across institutions and improve health care. For example, genome-wide association studies (GWAS) based on a large number of samples can identify disease-causing genetic variants. The privacy concern, however, has become a major hurdle for data management and utilization. Homomorphic encryption is one of the most powerful cryptographic primitives which can address the privacy and security issues. It supports the computation on encrypted data, so that we can aggregate data and perform an arbitrary computation on an untrusted cloud environment without the leakage of sensitive information.

**Methods:**

This paper presents a secure outsourcing solution to assess logistic regression models for quantitative traits to test their associations with genotypes. We adapt the semi-parallel training method by Sikorska et al., which builds a logistic regression model for covariates, followed by one-step parallelizable regressions on all individual single nucleotide polymorphisms (SNPs). In addition, we modify our underlying approximate homomorphic encryption scheme for performance improvement.

**Results:**

We evaluated the performance of our solution through experiments on real-world dataset. It achieves the best performance of homomorphic encryption system for GWAS analysis in terms of both complexity and accuracy. For example, given a dataset consisting of 245 samples, each of which has 10643 SNPs and 3 covariates, our algorithm takes about 43 seconds to perform logistic regression based genome wide association analysis over encryption.

**Conclusions:**

We demonstrate the feasibility and scalability of our solution.

## Background

Since National Institutes of Health (NIH) released the Gemonic Data Sharing policy allowing the use of cloud computing services for storage and analysis of controlled-access data [1], we are getting more challenge to ensure security and privacy of data in cloud computing systems. In the United States, the Health Insurance Portability and Accountability Act regulates medical care data sharing [2]. A community effort has been made to protect the privacy of genomic data, for example, iDASH (integrating Data for Analysis, Anonymization, Sharing) has hosted secure genome analysis competition for the past 5 years. This contest has encouraged cryptography experts to develop practical yet rigorous solutions for privacy preserving genomic data analysis. As a result, we could demonstrate the feasibility of secure genome data analysis using various cryptographic primitives such as homomorphic encryption (HE), differential privacy, multi-party computation, and software guard extension. In particular, HE has emerged as one of the promising solutions for secure outsourced computation over genomic data in practical biomedical applications [3–6].

### Summary of results

In this work, we provide a solution for the second track of iDASH 2018 competition, which aims to develop a method for outsourcing computation of Genome Wide Association Studies (GWAS) on homomorphically encrypted data. We propose a practical protocol to assess logistic regression model to compute *p*-values of different single nucleotide polymorphisms (SNPs). We investigate the association of genotypes and phenotypes by adjusting the models on the basis of covariates. The results will be used for identifying genetic variants that are statistically correlated with phenotypes of interest.

One year ago, participants of the third task in iDASH 2017 competition were challenged to train a single logistic regression model on encrypted data. Although significant performance improvements over existing solutions have been demonstrated [7, 8], it is still computationally intensive to perform logistic regression based GWAS. A straightforward implementation would require building one model for each SNP, incurring a high performance overhead of secure computation. This motivates the use of the semi-parallel algorithm, which was previously discussed in [9, 10]. Following the approach, our algorithm proceeds in two steps over encrypted data: (1) construct a logistic regression model by applying the gradient descent method of [7] while taking only the covariates into account, (2) compute the regression parameters of logistic regression corresponding to SNPs with one additional update of Newton’s method. The model in the first step can be computed very efficiently and can be used for all SNPs in the subsequent step. In the second step, we apply various techniques to enable computing the logistic regression updates for all SNPs in many parallel sub-steps. This approach enables us to obtain logistic regression based models for thousands of SNPs all in one.

Our solution is based on a homomorphic scheme by Cheon et al. [11] with support for approximate fixed-point arithmetic over the real numbers. Recently, a significant performance improvement was made in [8] based on the Residue Number System (RNS). The authors modified homomorphic operations so that they do not require any expensive RNS conversions. In this paper, we propose another RNS variant of approximate HE scheme which has some advantages for this task. Specifically, we adapt a different key-switching method which is a core operation in homomorphic multiplication or permutation. The earlier studies [8, 11] were based on the key-switching technique of [12] which introduces a special modulus. A special modulus had approximately the same bit-size as a ciphertext modulus to reduce the noise of key-switching procedure, but we observed that it is not the best option when the depth of an HE scheme is small. Instead, we combine the special modulus technique with RNS-friendly decomposition method [13]. As a result, we could minimize the parameter and thereby improve the performance while guaranteeing the same security level. We further leverage efficient packing techniques and parallelization approaches to reduce the storage requirement and running time.

### Related works

There are a number of recent research articles on HE-based machine learning applications. Kim et al. presented the first secure outsourcing method to train a logistic regression model on encrypted data [14] and the follow-up showed remarkably good performance with real data [7, 8]. For example, the training of a logistic regression model took about 3.6 minutes on encrypted data consisting of 1579 samples and 18 features. A slightly different approach is taken in [15], where the authors use Gentry’s bootstrapping technique in fully homomorphic encryption, so that their solution can run for an arbitrary number of iterations of gradient descent algorithm.

## Methods

The binary logarithm will be simply denoted by log(·). We denote vectors in bold, e.g. **a**, and matrices in upper-case bold, e.g. **A**. For an *n*×*m* matrix **A**, we use **A**_*i*_ to denote the *i*-th row of **A**, and **a**_*j*_ the *j*-th column of **A**. For a *d*_1_×*d* matrix **A**_1_ and a *d*_2_×*d* matrix **A**_2_,(**A**_1_;**A**_2_) denotes the (*d*_1_+*d*_2_)×*d* matrix obtained by concatenating two matrices in a vertical direction. If two matrices **A**_1_ and **A**_2_ have the same number of rows, (**A**_1_|**A**_2_) denotes a matrix formed by horizontal concatenation. We let *λ* denote the security parameter throughout the paper: all known valid attacks against the cryptographic scheme under scope should take *Ω*(2^*λ*^) bit operations.

### Logistic regression

Logistic regression is a widely used in statistical model when the response variable is categorical with two possible outcomes [16]. In particular, it is very popular in biomedical informatics research and serve as the foundation of many risk calculators [17–19].

Let the observed phenotype be given as a vector **y**∈{±1}^*n*^ of length *n*, the states of *p* many SNPs as the *n*×*p* matrix **S**, and the states of *k* many covariates as the *n*×*k* matrix **X**. Suppose that an intercept is included in the matrix of covariates, that is, **X** contains a column of ones. For convenience, let $\mathbf {u}_{i} = (\mathbf {X}_{i}, s_{ij}) \in \mathbb {R}^{k+1} $ for $i=1,\dots,n$. For each *j*∈[*p*], logistic regression aims to find an optimal vector ${\boldsymbol {\beta }} \in \mathbb {R}^{k+1}$ which maximizes the likelihood estimator $\prod _{i=1}^{n} \Pr [y_{i}|\mathbf {u}_{i}] = \prod _{i=1}^{n} \sigma (-y_{i} \cdot \mathbf {u}_{i}^{T}{\boldsymbol {\beta }}),$ where *σ*(*x*)=1/(1+ exp(−*x*)) is the sigmoid function, or equivalently minimizes the loss function, defined as the negative log-likelihood:

$$L({\boldsymbol{\beta}}) = \frac{1}{n}\sum_{i=1}^{n} \log\left(1 + \exp\left(-y_{i} \cdot \mathbf{u}_{i}^{T} {\boldsymbol{\beta}}\right)\right). $$

Note that ***β***=(***β***_**X**_|*β*_*j*_) depends on the index *j*, and we are particularly interested in the last component *β*_*j*_ that corresponds to the *j*-th SNP.

There is no closed form formula for the regression coefficients that minimizes the loss function. Instead, we employ an iterative process: we begin with some initial guess for the parameters and then repeatedly update them to make the loss smaller until the process converges. Specifically, the gradient descent (GD) takes a step in the direction of the steepest decrease of *L*. The method of GD can face a problem of zig-zagging along a local optima and this behavior of the method becomes typical if it increases the number of variables of an objective function. We can employ Nesterov’s accelerated gradient [20] to address this phenomenon, which uses moving average on the update vector and evaluates the gradient at this looked-ahead position.

#### Newton’s method

We can alternatively use Newton algorithm to estimate parameters [21]. It can be achieved by calculating the first and the second derivatives of the loss function, followed by the update: ${\boldsymbol {\beta }} \leftarrow {\boldsymbol {\beta }} - \left (\nabla ^{2}_{\small {\boldsymbol {\beta }}} L({\boldsymbol {\beta }})\right)^{-1} \cdot \nabla _{\small {\boldsymbol {\beta }}} L({\boldsymbol {\beta }})$. Let $p_{i} = \sigma \left (\mathbf {u}_{i}^{T} {\boldsymbol {\beta }}\right)$ for *i*∈[*n*]; then *p*_*i*_ represents the probability of success for each sample. We see that ∇_***β***_*L*(***β***)=**U**^*T*^(**y**−**p**) and $ \nabla ^{2}_{\small {\boldsymbol {\beta }}} L({\boldsymbol {\beta }}) = - \mathbf {U}^{T} \mathbf {W} \mathbf {U}$, where **U** is an *n*×(*k*+1) regressor matrix whose *i*-th row contains the variables $\mathbf {u}_{i}, \mathbf {p}= (p_{i})_{i=1}^{n}$ is a column vector of the estimated probabilities *p*_*i*_, and **W** is a diagonal weighting matrix with elements *w*_*i*_=*p*_*i*_(1− *p*_*i*_). Then the above update formula can be rewritten as

$$\begin{array}{*{20}l} {\boldsymbol{\beta}} & \leftarrow \left(\mathbf{U}^{T} \mathbf{W} \mathbf{U}\right)^{-1} \cdot \left(\mathbf{U}^{T} \mathbf{W} \mathbf{z}\right) \end{array} $$

where **z**=**U*β***+**W**^−1^(**y**−**p**). Here, the vector **z** is known as the *working response*. This method is also called *Iteratively Reweighted Least Squares*. More details can be found in [21]. On the other hand, the Fisher information **U**^*T*^**WU** can be partitioned into a block form:


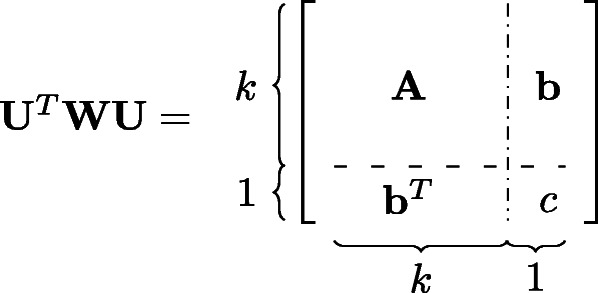


where $\mathbf {A} = \mathbf {X}^{T} \mathbf {W} \mathbf {X}, {\mathbf {s}}_{j} = (s_{ij})_{i=1}^{n}$ is a column vector of all samples of the *j*-th SNP, **b**=**X**^*T*^**Ws**_*j*_, and $c = {\mathbf {s}}_{j}^{T} \mathbf {W} {\mathbf {s}}_{j}$. Then the inverse of **U**^*T*^**WU** is


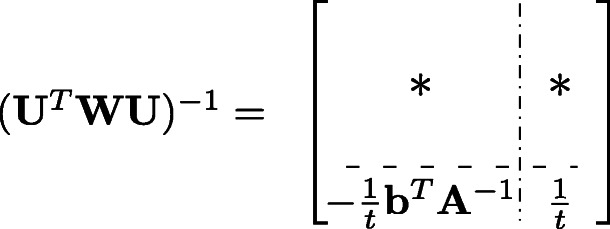


where *t*=*c*−**b**^*T*^**A**^−1^**b**. Therefore, the estimated SNP effect *β*_*j*_ and the variance for the estimation are computed by

1 $$\begin{array}{*{20}l} \beta_{j} &= -\frac{1}{t} \cdot \left(\mathbf{b}^{T} \mathbf{A}^{-1}\right) \cdot \left(\mathbf{X}^{T} \mathbf{W} \mathbf{z}\right) + \frac{1}{t} \cdot \left({\mathbf{s}}_{j}^{T} \mathbf{W} \mathbf{z}\right) \\ & = \frac{|\mathbf{A}| \cdot {\mathbf{s}}_{j}^{T} \mathbf{W} \mathbf{z} - \mathbf{b}^{T} \cdot \texttt{adj}(\mathbf{A})\cdot \left(\mathbf{X}^{T} \mathbf{W} \mathbf{z}\right)} {|\mathbf{A}| \cdot c - \mathbf{b}^{T} \cdot \texttt{adj}(\mathbf{A})\cdot \mathbf{b}},~ \end{array} $$

2 $$\begin{array}{*{20}l} \texttt{var}_{j} &= \frac{1}{c - \mathbf{b}^{T} \cdot \mathbf{A}^{-1}\cdot \mathbf{b}},  \end{array} $$

where adj(**A**) denotes the adjugate matrix and |**A**| the determinant of **A**.

### Full RNS variant of HEAAN, revisited

We apply the full RNS variant of the HEAAN scheme [11], called RNS-HEAAN [8], for efficient arithmetic over the real numbers. In addition, we modify some algorithms to meet our goals.

The previous RNS-HEAAN scheme uses some approximate modulus switching algorithms for the key-switching procedure. The evaluation key should have a much larger modulus compared to encrypted data due to multiplicative noise. In this work, we developed and implemented a new key-switching algorithm which provides a trade-off between complexity and parameter. Our new key-switching process requires more Number Theoretic Transformation (NTT) conversions, but the HE parameters such as the ring dimension *N* can be reduced while keeping the same security level. In particular, our method is more efficient than the previous one when the depth of a circuit to be evaluated is small.

The following is a simple description of RNS-HEAAN based on the ring learning with errors (RLWE) problem. Let $R=\mathbb {Z}[X]/(X^{N}+1)$ be a cyclotomic ring for a power-of-two integer *N*. An ordinary ciphertext of RNS-HEAAN can be represented as a linear polynomial *c*(*Y*)=*c*_0_+*c*_1_·*Y* over the ring *R*_*Q*_ where *Q* denotes the ciphertext modulus and *R*_*Q*_=*R* (mod *Q*) is the residue ring modulo *Q*.

$\bullet ~\underline {\texttt {Setup}(q,L,\eta ;1^{\lambda })}.$ Given a base integer module *q*, a maximum level *L* of computation, a bit precision *η*, and a security parameter *λ*, the Setup algorithm generates the following parameters:

- Choose a basis $\mathcal {D}= \{p_{0},q_{0}, q_{1},\ldots,q_{L}\}$ such that *q*_*i*_/*q*∈(1−2^−*η*^,1+2^−*η*^) for 1≤*i*≤*L*. We write $Q_{\ell } = \prod _{i=0}^{\ell } q_{i}$ for 0≤*ℓ*≤*L*.
- Choose a power-of-two integer *N*.
- Choose a secret key distribution *χ*_key_, an encryption key distribution *χ*_enc_, and an error distribution *χ*_err_ over *R*.

We always use the RNS form with respect to the basis $\{p_{0},q_{0},\dots,q_{\ell }\}$ (or its sub-basis) to represent polynomials in our scheme. For example, an element *a*(*X*) of $R_{Q_{\ell }}$ is identified with the tuple $(a_{0},a_{1},\dots,a_{\ell }) \in \prod _{i=0}^{\ell } R_{q_{i}}$ where *a*_*i*_=*a* (mod *q*_*i*_). We point out that all algorithms in our scheme are RNS-friendly, so that we do not have to perform any RNS conversions.

The main difference of our scheme from previous work [8] is that the key-switching procedure is based on both the decomposition and modulus raising techniques. The use of decomposition allows us to use a smaller parameter, but its complexity may be increased when the level of HE scheme is large. However, we realize that the GWAS analysis does not require a huge depth, so this new key-switching technique is beneficial to obtain a better performance in this specific application. The generation of switching key and key-switching algorithms are described as follows.

$\bullet ~\underline {\texttt {KSGen}(s_{1}, s_{2})}$. Given two secret polynomials *s*_1_,*s*_2_∈*R*, sample $\tilde a_{i}(X) \leftarrow U(R_{p_{0}\cdot Q_{L}})$ and errors $\tilde {e}_{i} \leftarrow \chi _{\text {err}}$ for 0≤*i*≤*L*. Output the switching key $\mathsf {swk}=\{\mathsf {swk}_{i}= (\tilde b_{i}, \tilde a_{i})\}_{0\le i\le L}\in \left (R_{p_{0} Q_{L}}^{2}\right)^{L+1}$ where $\tilde b_{i}=-\tilde a_{i}\cdot s_{2}+\tilde e_{i}+p_{0} B_{i}\cdot s_{1} \pmod {p_{0}\cdot Q_{L}}$ for the integer $B_{i}\in \mathbb {Z}_{Q_{L}}$ such that *B*_*i*_=1 (mod *q*_*i*_) and *B*_*i*_=0 (mod *q*_*j*_) for all *j*≠*i*.

$\bullet ~\underline {\texttt {KeySwitch}_{\mathsf {swk}}(\mathsf {ct})}$. For $\mathsf {ct}=(c_{0}, c_{1})\in R_{Q_{\ell }}^{2}$, let *c*_1,*i*_=*c*_1_ (mod *q*_*i*_) for 0≤*i*≤*ℓ*. We first compute $\tilde {\mathsf {ct}}=\sum _{i=0}^{\ell } c_{1,i}\cdot \mathsf {swk}_{i} \pmod {p_{0} Q_{\ell }},$ and then return the ciphertext $\mathsf {ct}'=(c_{0}, 0)+\lfloor {p_{0}^{-1}\cdot \tilde {\mathsf {ct}}}\rceil \pmod {Q_{\ell }}$.

The idea of key-switching procedure is used to relinearize a ciphertext in homomorphic multiplication algorithm below. All other algorithms including key generation, encryption and decryption are exactly same as the previous RNS-based scheme.

$\bullet ~\underline {\texttt {KeyGen}(1^{\lambda })}.$

- Sample *s*←*χ*_key_ and set the secret key as *sk*=(1,*s*).
- Sample $a\leftarrow U(R_{Q_{L}})$ and *e*←*χ*_err_. Set the public key *pk* as $\mathsf {pk} =(b,a) \in R^{2}_{Q_{L}}$ where *b*=−*a*·*s*+*e* (mod *Q*_*L*_).
- Set the evaluation key as *evk*←KSGen(*s*^2^,*s*).

$\bullet ~\underline {\texttt {Enc}_{\mathsf {pk}}(m)}$. Given *m*∈*R*, sample *v*←*χ*_enc_ and *e*_0_,*e*_1_←*χ*_err_. Output the ciphertext *ct*=*v*·*pk*+(*m*+*e*_0_,*e*_1_) (mod *Q*_*L*_).

$\bullet ~\underline {\texttt {Dec}_{\mathsf {sk}}({\mathsf {ct}})}$. Given ciphertext ${\mathsf {ct}} \in R_{Q_\ell }^{2}$, output 〈*ct*,*sk*〉 (mod *q*_0_).

$\bullet ~\underline {\texttt {Add}({\mathsf {ct}},{\mathsf {ct}}')}$. Given two ciphertexts ${\mathsf {ct}},{\mathsf {ct}}'\in R_{Q_{\ell }}^{2}$, output the ciphertext *ct*_*add*_=*ct*+*ct*^′^ (mod *Q*_*ℓ*_).

$\bullet ~\underline {\texttt {Mult}_{\mathsf {evk}}({\mathsf {ct}},{\mathsf {ct}}')}$. For two ciphertexts *ct*=(*c*_0_,*c*_1_) and *ct*^′^=(*c*0′,*c*1′), compute *d*_0_=*c*_0_*c*0′,*d*_1_=*c*_0_*c*1′+*c*0′*c*_1_,*d*_2_=*c*_1_*c*1′ (mod *Q*_*ℓ*_). Let *c*_2,*i*_=*d*_2_ (mod *q*_*i*_) for 0≤*i*≤*ℓ*, and compute $\tilde {\mathsf {ct}}=\sum _{i=0}^{\ell } c_{2,i}\cdot \mathsf {evk}_{i} \pmod {p_{0} Q_{\ell }}$. Output the ciphertext ${\mathsf {ct}}'=(c_{0}, c_{1})+\lfloor {p_{0}^{-1}\cdot \tilde {\mathsf {ct}}}\rceil \pmod {Q_{\ell }}$.

Finally, RNS-HEAAN provides the rescaling operation to round messages over encryption, thereby enabling to control the magnitude of messages during computation.

$\bullet ~\underline {\texttt {ReScale}({\mathsf {ct}})}$. For given ${\mathsf {ct}}\in \mathcal {R}_{Q_{\ell }}^{2}$, return the ciphertext ${\mathsf {ct}}'=\lfloor {q_{\ell }^{-1}\cdot {\mathsf {ct}}}\rceil \pmod {Q_{\ell -1}}$.

It is a common practice to rescale the encrypted message after each multiplication as we round-off the significant digits after multiplication in plain fixed/floating point computation. In the next section, we assume that the rescaling procedure is included in homomorphic multiplications for simpler description, but a rigorous analysis about level consumption will be provided later in the parameter setting section.

As in the original HEAAN scheme, the native plaintext space can be understood as an *N*/2-dimensional complex vector space (each vector component is called a *plaintext slot*). Addition and multiplication in *R* correspond to component-wise addition and multiplication on plaintext slots. Furthermore, it provides an operation that shifts the plaintext vector over encryption. For a ciphertext *ct* encrypting a plaintext vector $(m_{1},\ldots,m_{\ell }) \in \mathbb {R}^{\ell }$, we could obtain an encryption of a shifted vector (*m*_*r*+1_,…,*m*_*ℓ*_,*m*_1_,…,*m*_*r*_). Let us denote such operation by *Rot*(*ct*;*r*). For more detail, we refer the reader to [8]. In the rest of this paper, we let *N*_2_=*N*/2 and denote by E(·) the encryption function for convenience.

### Database encoding

As noted before, the learning data are recorded into an *n*×*k* matrix **X** of covariates, an *n*×*p* binary matrix **S**=(*s*_*ij*_) of all the SNP data, and an *n*-dimensional binary column vector **y** of the dependent variable. In large-scale GWAS, the number of parameters of SNPs, *p* can be in the thousands, so we split the SNP data into several *N*_2_-dimensional vectors, encrypt them, and send the resulting ciphertexts to the server. For simplicity, we assume in the following discussion that each row of **S** is encrypted into a single ciphertext. More specifically, for 1≤*i*≤*n* and for 1≤*ℓ*≤*k*, we encrypt E(*x*_*iℓ*_**S**_*i*_)=E(*x*_*iℓ*_*s*_*i*1_,…,*x*_*iℓ*_*s*_*ip*_). As mentioned before, we add a column of ones to **X** to allow for an intercept in the regression; that is, we assume *x*_*i*1_=1 for all 1≤*i*≤*n*. So, when *ℓ*=1, the ciphertext E(*x*_*i*1_**S**_*i*_) encrypts exactly the *i*-th SNP sample.

Next, consider the matrix $\mathbf {y}^{T} \mathbf {X} \in \mathbb {R}^{n \times k}$ defined as

$$\begin{array}{*{20}l} {\mathbf{y}^{T} \mathbf{X}} & = \left[\begin{array}{ccc} y_{1} \mathbf{X}_{1}; & \cdots & ;y_{n} \mathbf{X}_{n} \end{array}\right] \\ & = \left[\begin{array}{cccc} y_{1} x_{11} & y_{1} x_{12} & \cdots & y_{1} x_{1k} \\ y_{2} x_{21} & y_{2} x_{22} & \cdots & y_{2} x_{2k} \\ \vdots & \vdots & \ddots & \vdots \\ y_{n} x_{n1} & y_{n} x_{n2} & \cdots & y_{n} x_{nk} \end{array}\right]. \end{array} $$

For simplicity, we assume that *n* and *k* are power-of-two integers satisfying log*n*+ log*k*≤ log(*N*_2_). Kim et al. [7] suggested an efficient encoding map to encode the whole matrix **y**^*T*^**X** in a single ciphertext in a row-by-row manner. Specifically, we will identify this matrix with a vector in $\mathbb {R}^{n\cdot k}$, that is,

$$\begin{array}{*{20}l} {\mathbf{y}^{T} \mathbf{X}} & \mapsto (y_{1} \mathbf{X}_{1}| \cdots| y_{n} \mathbf{X}_{n}) \\ & = (y_{1}x_{11},\dots,y_{1}x_{1k},\dots,y_{n}x_{n1},\dots,y_{n} x_{nk}). \end{array} $$

Similarly, we identify the matrix **X** with a vector in $\mathbb {R}^{n\cdot k}$ as follows:

$${\mathbf{X}} \mapsto (\mathbf{X}_{1}| \cdots| \mathbf{X}_{n}) = (x_{11},\dots,x_{1k},\dots,x_{n1},\dots,x_{nk}).$$

For an efficient implementation, we can make *N*_2_/(*k*·*n*) copies of each component of **y**^*T*^**X** and **X** to encode them into fully packed plaintext slots. For example, we can generate the encryption of **y**^*T*^**X** as

$$\texttt{E}({\mathbf{y}^{T} \mathbf{X}})= \texttt{E}(y_{1}\mathbf{X}_{1}^{(N_2/(k \cdot n))}|\cdots| y_{n}\mathbf{X}_{n}^{(N_2/(k \cdot n))}), $$

where $y_{i}\mathbf {X}_{i}^{(N_2/(k \cdot n))}$ denotes an array containing *N*_2_/(*k*·*n*) copies of *y*_*i*_**X**_*i*_. In the case of the target vector **y**, we make *N*_2_/*n* copies of each entry, so that the encoding aligns *y*_*i*_ with each copies of *y*_*i*_**X**_*i*_ and **X**_*i*_ in the ciphertexts. Let us denote the generated ciphertext by E(**y**).

Finally, we now consider how to encrypt the covariance matrix **X**^*T*^**X** which can be used for computing the adjugate matrix and determinant of **A**=**X**^*T*^**WX**. The adjugate adj(**A**) is a *k*×*k* matrix whose entries are defined as $\texttt {adj}(\mathbf {A})_{j\ell } := (-1)^{j+\ell }\cdot |\hat {\mathbf {A}}_{\ell j}|$ for 1≤*j*,*ℓ*≤*k*, where $|\hat {\mathbf {A}}_{\ell j}|$ is the determinant of $\hat {\mathbf {A}}_{\ell j}$. Here, $\hat {\mathbf {A}}_{\ell j}$ is a (*k*− 1)×(*k*−1) sub-matrix obtained by removing the *j*-th column and *ℓ*-th row from **A**. For example, when *k*=4, the determinant $|\hat {\mathbf {A}}_{11}|$ is computed by *a*_22_(*a*_33_*a*_44_−*a*_34_*a*_43_)+*a*_23_(*a*_34_*a*_42_−*a*_32_*a*_44_)+*a*_24_(*a*_32_*a*_43_−*a*_33_*a*_42_), which can be rewritten as a component-wise product of three vectors

$$\begin{array}{*{20}l} \mathbf{A}_{1,1,1} & = (a_{22},-a_{22},a_{23},-a_{23},a_{24},-a_{24}), \\ \mathbf{A}_{1,1,2} & = (a_{33},-a_{34},a_{34},-a_{32},a_{32},-a_{33}), \\ \mathbf{A}_{1,1,3} & = (a_{44},-a_{43},a_{42},-a_{44},a_{43},-a_{42}). \end{array} $$

In general, we can consider (*k*−1)!-dimensional vectors **A**_*j*,*ℓ*,1_,**A**_*j*,*ℓ*,2_,…,**A**_*j*,*ℓ*,(*k*−1)_ that can be used to compute $|\hat {\mathbf {A}}_{\ell j}|$. To do so, for each *i*∈[*n*], we first pre-compute the *i*-th covariance matrix $\mathbf {X}_{i}^{T} \mathbf {X}_{i} \in \mathbb {R}^{k\times k}$ and generate the corresponding vector $\left (\mathbf {X}_{i}^{T} \mathbf {X}_{i}\right)_{j,\ell,t}$ for 1≤*j*≤*ℓ*≤*k* and 1≤*t*≤*k*−1. Suppose that *N*_2_≥*n*·(*k*−1)!. Let *ϕ*=*N*_2_/(*n*·(*k*−1)!), and we encrypt the following concatenated vector

$$\Sigma_{j,\ell,t} = \left((\mathbf{X}_{1}^{T} \mathbf{X}_{1})_{j,\ell,t}^{(\phi)} \ | \ \ldots \ | (\mathbf{X}_{n}^{T} \mathbf{X}_{n})_{j,\ell,t}^{(\phi)} \right).$$

We denote the resulting ciphertext by E(*Σ*_*j*,*ℓ*,*t*_).

An alternative choice is to encrypt SNPs, covariates, and phenotype vectors in a separate way. The server can reconstruct the aforementioned encryptions by applying homomorphic operations, but it requires additional levels for the computation. So, we used the former encryption algorithm in the implementation, thereby saving on the depth and time in the evaluation. Our encoding system has another advantage, in that it can be applied to *horizontally partitioned* data where each party has a subset of the rows in dataset. In this case, each party encrypts their locally computed quantities on their data and sends them to the server. Then the server aggregates them to obtain encryptions of the shared data as the ones in our encryption method.

### Homomorphic evaluation of logistic regression

The main idea of the semi-parallel logistic regression analysis [9, 10] is to assume that the probabilities predicted by a model without SNP will not change much once SNP is included to the model. We will follow their approach, where the first step is to construct a logistic regression model taking only the covariates into account, and the second step is to compute the model coefficients of the logistic regression corresponding to the SNP in a semi-parallel way.

We start with a useful aggregation operation across plaintext slots from the literature [22–24]. This algorithm is referred as AllSum, which is parameterized by integers *ψ* and *α*. See Algorithm 1 for an implementation. Let *ℓ*=*ψ*·*α*. Given a ciphertext *ct* representing a plaintext vector $\mathbf {m}=(m_{1},\ldots,m_{\ell })\in \mathbb {R}^{\ell }$, the AllSum algorithm outputs a ciphertext *ct*^′^ encrypting

$$\begin{array}{*{20}l} ' &= \left(\sum_{j=0}^{\alpha-1} m_{\psi j+1},\sum_{j=0}^{\alpha-1} m_{\psi j+2},\ldots \sum_{j=0}^{\alpha-1} m_{\psi (j+1)},\right.\\ & \left.\sum_{j=0}^{\alpha-1} m_{\psi j+1},\sum_{j=0}^{\alpha-1} m_{\psi j+2},\ldots \sum_{j=0}^{\alpha-1} m_{\psi (j+1)},\ldots\right), \end{array} $$

i.e., $\mathbf {m}'_{i} = \sum _{j=0}^{\alpha -1} \mathbf {m}_{\psi j+i}$ for 1≤*i*≤*ψ*, and **m***ψj*+*i*′=**m***i*′ for 1≤*j*≤*α*−1. For example, when *ψ*=1, it returns an encryption of the sum of the elements of **m**.


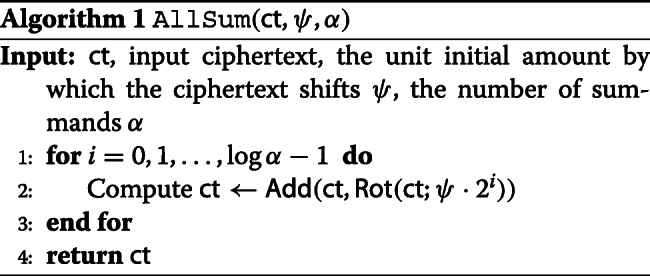


As mentioned before, our algorithm consists of two steps to perform the semi-parallel logistic regression training while taking as input the following ciphertexts: {E(*x*_*iℓ*_**S**_*i*_)},E(**y**^*T*^**X**),E(**X**),E(**y**), and {E(*Σ*_*j*,*ℓ*,*t*_)}, for 1≤*i*≤*n*,1≤*j*≤*ℓ*≤*k*, and 1≤*t*≤*k*−1.

#### Logistic regression model training for covariates

The best solution to train a logistic regression model from homomorphically encrypted dataset is to evaluate Nesterov’s accelerated gradient descent method [7, 8]. We adapt their evaluation strategy to train a model for covariates.

Step 0: For simplicity, let **v**_*i*_=*y*_*i*_**X**_*i*_ and *ℓ*=*N*_2_/(*k*·*n*). Since the input ciphertext E(**y**^*T*^**X**) represents *ℓ* copies of **v**_*i*_, Step 6 in [7] outputs the following ciphertext that encrypts the same number of copies of the vectors $\sigma (\mathbf {v}_{i}^{T}\boldsymbol {\beta }_{\mathbf {X}})\cdot \mathbf {v}_{i}$:

$${\mathsf{ct}}_{6} = \texttt{E}\left[\begin{array}{ccc} \sigma(\mathbf{v}_{1}^{T}\boldsymbol{\beta}_{\mathbf{X}})\cdot v_{11} & \cdots & \sigma(\mathbf{v}_{1}^{T} \boldsymbol{\beta}_{\mathbf{X}})\cdot v_{1k} \\ \vdots & \ddots & \vdots \\ \sigma(\mathbf{v}_{1}^{T}\boldsymbol{\beta}_{\mathbf{X}})\cdot v_{11} & \cdots & \sigma(\mathbf{v}_{1}^{T} \boldsymbol{\beta}_{\mathbf{X}})\cdot v_{1k} \\ \vdots & \ddots & \vdots \\ \sigma(\mathbf{v}_{n}^{T}\boldsymbol{\beta}_{\mathbf{X}})\cdot v_{n1} & \cdots & \sigma(\mathbf{v}_{n}^{T} \boldsymbol{\beta}_{\mathbf{X}})\cdot v_{nk} \\ \vdots & \ddots & \vdots \\ \sigma(\mathbf{v}_{n}^{T}\boldsymbol{\beta}_{\mathbf{X}})\cdot v_{n1} & \cdots & \sigma(\mathbf{v}_{n}^{T} \boldsymbol{\beta}_{\mathbf{X}})\cdot v_{nk} \\ \end{array}\right].$$

Then Step 7 in [7] is changed from AllSum(*ct*_6_,*k*,*n*) into *ct*_7_=AllSum(*ct*_6_,*N*_2_/*n*,*n*), so that the output ciphertext is as follows:

$${\mathsf{ct}}_{7} = \texttt{E}\left[\begin{array}{ccc} \sum_{i} \sigma(\mathbf{v}_{i}^{T}\boldsymbol{\beta}_{\mathbf{X}})\cdot v_{i1} & \cdots & \sum_{i} \sigma(\mathbf{v}_{i}^{T}\boldsymbol{\beta}_{\mathbf{X}})\cdot v_{ik} \\ \sum_{i} \sigma(\mathbf{v}_{i}^{T}\boldsymbol{\beta}_{\mathbf{X}})\cdot v_{i1} & \cdots & \sum_{i} \sigma(\mathbf{v}_{i}^{T}\boldsymbol{\beta}_{\mathbf{X}})\cdot v_{ik} \\ \vdots & \ddots & \vdots \\ \sum_{i} \sigma(\mathbf{v}_{i}^{T}\boldsymbol{\beta}_{\mathbf{X}})\cdot v_{i1} & \cdots & \sum_{i} \sigma(\mathbf{v}_{i}^{T}\boldsymbol{\beta}_{\mathbf{X}})\cdot v_{ik} \\ \end{array}\right].$$

In the end, the model parameters ***β***_**X**_ are encrypted as a ciphertext with fully-packed plaintext slots. More precisely, it yields encrypted model parameters E(***β***_**X**_) that represent a plaintext vector containing *N*_2_/*k*=*ℓ*·*n* copies of ***β***_**X**_ as follows:

$$\texttt{E}(\boldsymbol{\beta}_{\mathbf{X}}) =\left[\begin{array}{cccc} {\beta_{X}}_{1} & {\beta_{X}}_{2} & \cdots & {\beta_{X}}_{k} \\ {\beta_{X}}_{1} & {\beta_{X}}_{2} & \cdots & {\beta_{X}}_{k} \\ \vdots & \vdots & \ddots & \vdots \\ {\beta_{X}}_{1} & {\beta_{X}}_{2} & \cdots & {\beta_{X}}_{k} \\ \end{array}\right].$$

#### Parallel logistic regression model building for SNPs

Starting with $\boldsymbol {\beta } = (\boldsymbol {\beta }_{\mathbf {X}}, 0) \in \mathbb {R}^{k+1}$, we will perform one step of Newton’s method for regression with SNPs. This implies that the regression coefficients multiplied by the values of the predictor are **U*β***=**X*β***_**X**_, so for all *i*∈[*n*], if we let the predicted value be $\hat {y}_{i} = \mathbf {u}_{i}^{T} {\boldsymbol {\beta }} $, then we have $\hat {y}_{i} = {\mathbf {x}}_{i}^{T} {\boldsymbol {\beta }}_{\mathbf {X}}$. We note that

3 $$\begin{array}{*{20}l} (\mathbf{W} \mathbf{z})_{i} & = w_{i} \cdot z_{i} = p_{i}(1-p_{i}) \cdot \hat{y}_{i} + (y_{i}- p_{i})  \end{array} $$

with $p_{i} = \sigma (\hat {y}_{i})$. In the following, we describe how to securely evaluate these variables from the model parameters ***β***_**X**_. In the end, the server outputs encryptions of the numerator and the denominator of Eq. 1, denoted by $\beta _{j}^{\star }$ and $\beta _{j}^{\dagger }$.

Step 1: Let $\hat {\mathbf {y}} = (\hat {y}_{i})_{i=1}^{n}$ be a column vector of the predicted values. The goal of this step is to generate its encryption. The server first performs homomorphic multiplication between two ciphertexts E(***β***_**X**_) and E(**X**), and then applies AllSum to the resulting ciphertext:

4 $$ \texttt{E}(\hat{\mathbf{y}}_{\star}) \leftarrow \texttt{AllSum}(\texttt{E}(\boldsymbol{\beta}_{\mathbf{X}}) \cdot \texttt{E}(\mathbf{X}), 1, k).  $$

The output ciphertext $\texttt {E}(\hat {\mathbf {y}}_{\star })$ encrypts the values $\hat {y}_{i}$ at (*t*·*k*+1) positions for (*i*−1)·*ℓ*≤*t*<*i*·*ℓ* and some garbage values in the other entries, denoted by ⋆, i.e.,

$$\texttt{E}(\hat{\mathbf{y}}_{\star})=\texttt{E}\left[\begin{array}{cccc} \hat{y}_{1} & \star & \cdots & \star \\ \vdots & \vdots & \ddots & \vdots \\ \hat{y}_{1} & \star & \cdots & \star \\ \vdots & \vdots & \ddots & \vdots \\ \hat{y}_{n} & \star & \cdots & \star \\ \vdots & \vdots & \ddots & \vdots \\ \hat{y}_{n} & \star & \cdots & \star \end{array}\right].$$

The server then performs a constant multiplication by *c* to annihilate the garbage values. The polynomial *c*←Encode(**C**) is the encoding of the following matrix, where Encode(·) is a standard procedure in [11] to encode a real vector as a ring element in *R*:

$$\mathbf{C}=\left[\begin{array}{cccc} 1 & 0 & \cdots & 0 \\ 1 & 0 & \cdots & 0 \\ \vdots & \vdots & \ddots & \vdots \\ 1 & 0 & \cdots & 0 \end{array}\right].$$

The next step is to replicate the values $\hat {y}_{i}$ to the other columns:

$$\texttt{E}(\hat{\mathbf{y}}) \leftarrow \texttt{AllSum}(\texttt{CMult}(\texttt{E}(\hat{\mathbf{y}}_{\star}); c), -1, k), $$

denoted by CMult(·) a scalar multiplication. So, the output ciphertext $\texttt {E}(\hat {\mathbf {y}})$ has *N*_2_/*n*=*ℓ*·*k* copies of $\hat {y_{i}}$:

$$\texttt{E}(\hat{\mathbf{y}})=\texttt{E}\left[\begin{array}{cccc} \hat{y}_{1} & \hat{y}_{1} & \cdots & \hat{y}_{1}\\ \vdots & \vdots & \ddots & \vdots \\ \hat{y}_{1} & \hat{y}_{1} & \cdots & \hat{y}_{1}\\ \vdots & \vdots & \ddots & \vdots \\ \hat{y}_{n} & \hat{y}_{n} & \cdots & \hat{y}_{n} \\ \vdots & \vdots & \ddots & \vdots \\ \hat{y}_{n} & \hat{y}_{n} & \cdots & \hat{y}_{n} \\ \end{array}\right].$$

Step 2: This step is simply to evaluate the approximating polynomial of the sigmoid function by applying the pure SIMD additions and multiplications:

$$\texttt{E}(\mathbf{p}) \leftarrow \sigma(\texttt{E}(\hat{\mathbf{y}})). $$

Then the server securely computes the weights *w*_*i*_ and carries out their multiplication with the working response vector **z** using Eq. 3:

5 $$\begin{array}{*{20}l} \texttt{E}(\mathbf{w}) & \leftarrow \texttt{E}(\mathbf{p}) \cdot (1 - \texttt{E}(\mathbf{p})),  \\ \texttt{E}(\mathbf{W}\mathbf{z}) & \leftarrow \texttt{E}(\mathbf{w}) \cdot \texttt{E}(\hat{\mathbf{y}}) + (\texttt{E}(\mathbf{y}) - \texttt{E}(\mathbf{p})). \end{array} $$

Here the two output ciphertexts containing *N*_2_/*n* copies of the values *w*_*i*_ and *w*_*i*_*z*_*i*_, respectively:

$$\texttt{E}({\mathbf{w}})=\texttt{E}\left[\begin{array}{cccc} w_{1} & w_{1} & \cdots & w_{1}\\ \vdots & \vdots & \ddots & \vdots \\ w_{1} & w_{1} & \cdots & w_{1}\\ \vdots & \vdots & \ddots & \vdots \\ w_{n} & w_{n} & \cdots & w_{n} \\ \vdots & \vdots & \ddots & \vdots \\ w_{n} & w_{n} & \cdots & w_{n} \\ \end{array}\right],$$

$$\texttt{E}({\mathbf{W}\mathbf{z}})=\texttt{E}\left[\begin{array}{cccc} w_{1} z_{1} & w_{1} z_{1} & \cdots & w_{1} z_{1}\\ \vdots & \vdots & \ddots & \vdots \\ w_{1} z_{1} & w_{1} z_{1} & \cdots & w_{1} z_{1}\\ \vdots & \vdots & \ddots & \vdots \\ w_{n} z_{n} & w_{n} z_{n} & \cdots & w_{n} z_{n}\\ \vdots & \vdots & \ddots & \vdots \\ w_{n} z_{n} & w_{n} z_{n} & \cdots & w_{n} z_{n}\\ \end{array}\right].$$

Step 3: The goal of this step is to generate trivial encryptions E(*w*_*i*_) such that for *i*∈[*n*],E(*w*_*i*_) has *w*_*i*_ in all positions of its plaintext vector. We employ the hybrid algorithm of [22] for replication, denoted by Replicate(·). The server outputs *n* ciphertexts

$$\{\texttt{E}(w_{i})\}_{1 \le i \le n} \leftarrow \texttt{Replicate}(\texttt{E}(\mathbf{w})). $$

Similarly, the server takes the ciphertext E(**Wz**) and performs another replication operation:

$$\{\texttt{E}(w_{i} z_{i})\}_{1 \le i \le n} \leftarrow \texttt{Replicate}(\texttt{E}(\mathbf{W}\mathbf{z})). $$

Step 4: For all *j*∈[*p*], we define the vector ${\mathbf {b}}_{j} = \mathbf {X}^{T} \mathbf {W} {\mathbf {s}}_{j} \in \mathbb {R}^{k}$ and denote the *ℓ*-th component of **b**_*j*_ by *b*_*jℓ*_. We note that $ b_{j\ell } = {\mathbf {x}}_{\ell }^{T} \mathbf {W} {\mathbf {s}}_{j} = \sum _{i=1}^{n} (x_{i\ell }\cdot w_{i} \cdot s_{ij}), $ where ${\mathbf {x}}_{\ell } = (x_{i\ell })_{i=1}^{n}$ is the *j*-th column of the design matrix **X**. Then, for all *ℓ*∈[*k*], the server generates encryptions of the vectors $\mathbf {B}_{\ell } = \mathbf {x}_{\ell }^{T} \mathbf {W} \mathbf {S} = (b_{1\ell },b_{2\ell },\ldots,b_{p\ell })$ by computing

6 $$ \texttt{E}(\mathbf{B}_{\ell}) \leftarrow \sum_{i=1}^{n} \texttt{E}(w_{i}) \cdot \texttt{E}(x_{i\ell} \mathbf{S}_{i}).  $$

On the other hand, since we add a column of ones to the matrix **X**, we have $ c_{j} = {\mathbf {s}}_{j}^{T} \mathbf {W} {\mathbf {s}}_{j} = \sum _{i=1}^{n} w_{i} \cdot s_{ij} = \sum _{i=1}^{n} x_{i1} \cdot w_{i} \cdot s_{ij} = b_{1j} $ for *j*∈[*p*], which implies that E(**B**_1_) can be understood as an encryption of (*c*_1_,*c*_2_,…,*c*_*p*_).

Step 5: This step is to securely compute the values ${\mathbf {s}}_{j}^{T} \mathbf {W} \mathbf {z} = \sum _{i=1}^{n} s_{ij} \cdot w_{i} \cdot z_{i}$ for *j*∈[*p*]. Specifically, the server performs the following computation:

7 $$ \texttt{E}\left({\mathbf{s}}_{1}^{T} \mathbf{W} \mathbf{z},\ldots,{\mathbf{s}}_{p}^{T} \mathbf{W} \mathbf{z}\right) \leftarrow \sum_{i=1}^{n} \texttt{E}(w_{i}z_{i}) \cdot \texttt{E}(x_{i1}\mathbf{S}_{i}).  $$

Step 6: The goal of this step is to securely compute the vector **X**^*T*^**Wz** such that the *ℓ*-th element is obtained by ${\mathbf {x}}_{\ell }^{T} \mathbf {W} \mathbf {z} = \sum _{i=1}^{n} (x_{i\ell }\cdot w_{i} \cdot z_{i})$ for *ℓ*∈[*k*]. The server first performs the pure SIMD multiplication between two ciphertexts E(**X**) and E(**Wz**):

8 $$  \texttt{E}(\mathbf{X}\odot \mathbf{W}\mathbf{z}) \leftarrow \texttt{E}(\mathbf{X}) \cdot \texttt{E}(\mathbf{W}\mathbf{z}).  $$

Here, the output ciphertext E(**X**⊙**Wz**) encrypts the values *x*_*iℓ*_*w*_*i*_*z*_*i*_:

$$\texttt{E}(\mathbf{X}\odot \mathbf{W}\mathbf{z}) = \texttt{E}\left[\begin{array}{cccc} x_{11} w_{1} z_{1} & x_{12} w_{1} z_{1} & \cdots & x_{1k} w_{1} z_{1} \\ \vdots & \vdots & \ddots & \vdots \\ x_{11} w_{1} z_{1} & x_{12} w_{1} z_{1} & \cdots & x_{1k} w_{1} z_{1} \\ \vdots & \vdots & \ddots & \vdots \\ x_{n1} w_{n} z_{n} & x_{n2} w_{n} z_{n} & \cdots & x_{nk} w_{n} z_{n} \\ \vdots & \vdots & \ddots & \vdots \\ x_{n1} w_{n} z_{n} & x_{n2} w_{n} z_{n} & \cdots & x_{nk} w_{n} z_{n} \\ \end{array}\right].$$

Then the server aggregates the values in the same column to obtain a ciphertext encrypting ${\mathbf {x}}_{\ell }^{T} \mathbf {W} \mathbf {z}$:

$$ \texttt{E}\left(\mathbf{X}^{T}\mathbf{W}\mathbf{z}\right) \leftarrow \texttt{AllSum}(\texttt{E}(\mathbf{X}\odot \mathbf{W}\mathbf{z})), N_2/(k \cdot n), n). $$

Notice that this ciphertext contains the scalar ${\mathbf {x}}_{\ell }^{T} \mathbf {W} \mathbf {z}$ in every entry of the *ℓ*-th column, for 1≤*ℓ*≤*k*:

$$\texttt{E}(\mathbf{X}^{T}\mathbf{W}\mathbf{z}) =\texttt{E}\left[\begin{array}{cccc} {\mathbf{x}}_{1}^{T} \mathbf{W} \mathbf{z} & {\mathbf{x}}_{2}^{T} \mathbf{W} \mathbf{z} & \cdots & {\mathbf{x}}_{k}^{T} \mathbf{W} \mathbf{z} \\ {\mathbf{x}}_{1}^{T} \mathbf{W} \mathbf{z} & {\mathbf{x}}_{2}^{T} \mathbf{W} \mathbf{z} & \cdots & {\mathbf{x}}_{k}^{T} \mathbf{W} \mathbf{z} \\ \vdots & \vdots & \ddots & \vdots \\ {\mathbf{x}}_{1}^{T} \mathbf{W} \mathbf{z} & {\mathbf{x}}_{2}^{T} \mathbf{W} \mathbf{z} & \cdots & {\mathbf{x}}_{k}^{T} \mathbf{W} \mathbf{z} \\ \end{array}\right].$$

Finally, it outputs *k* ciphertexts, each encrypting ${\mathbf {x}}_{\ell }^{T} \mathbf {W} \mathbf {z}$ for 1≤*ℓ*≤*k*, by applying the replication operation as follows:

$$\left\{\texttt{E}\left({\mathbf{x}}_{\ell}^{T} \mathbf{W} \mathbf{z}\right)\right\}_{1\le \ell \le k} \leftarrow \texttt{Replicate}(\texttt{E}(\mathbf{X}^{T}\mathbf{W}\mathbf{z})). $$

Step 7: The goal of this step is to compute the encryptions of the adjugate matrix and the determinant of **A**=**X**^*T*^**WX**. We note that

$$\begin{array}{*{20}l} \mathbf{A}_{r,s,t} & = \left(\sum_{i=1}^{n} w_{i} \cdot \mathbf{X}_{i}^{T} \mathbf{X}_{i}\right)_{r,s,t} = \sum_{i=1}^{n} w_{i} \cdot \left(\mathbf{X}_{i}^{T} \mathbf{X}_{i}\right)_{r,s,t}  \end{array} $$

for 1≤*r*≤*s*≤*k* and 1≤*t*≤*k*−1. The server first multiplies the ciphertexts E(*Σ*_*r*,*s*,*t*_) with the ciphertext E(**w**) to obtain

9 $$  \texttt{E}(\Sigma'_{r,s,t}) \leftarrow \texttt{E}(\mathbf{w}) \cdot \texttt{E}(\Sigma_{r,s,t}).  $$

Here, the ciphertext E(*Σr*,*s*,*t*′) encrypts *n* vectors $w_{i} \cdot (\mathbf {X}_{i}^{T} \mathbf {X}_{i})_{r,s,t}$ for 1≤*i*≤*n*. Then we apply AllSum to aggregate these vectors and obtain **A**_*r*,*s*,*t*_:

$$\texttt{E}(\mathbf{A}_{r,s,t}) \leftarrow \texttt{AllSum}(\texttt{E}(\Sigma'_{r,s,t}), \phi, n). $$

Next, the server performs multiplications between the ciphertexts E(**A**_*r*,*s*,*t*_) as follows:

10 $$ \texttt{E}(\Sigma_{r,s}) \leftarrow \prod_{t=1}^{k-1} \texttt{E}(\mathbf{A}_{r,s,t}).  $$

The adjugate matrix can be obtained by aggregating (*k*−1)! many values in E(*Σ*_*r*,*s*_):

$$\texttt{E}(\texttt{adj}(\mathbf{A})_{r,s}) \leftarrow \texttt{AllSum}(\texttt{E}(\Sigma_{r,s}),1,(k-1)!). $$

In addition, the server computes

$$\texttt{E}(x_{1r}\mathbf{W}) \leftarrow \texttt{AllSum}(\texttt{E}(x_{1r}) \cdot \texttt{E}(\mathbf{w}), N_2/n, n) $$

for 1≤*r*≤*k*, and obtains a trivial encryption of the determinant of **A** as follows:

$$\texttt{E}(|\mathbf{A}|) \leftarrow \sum_{r=1}^{k} \texttt{E}(x_{1r}\mathbf{W}) \cdot \texttt{E}(\texttt{adj}(\mathbf{A})_{1r}). $$

Step 8: The final step is to securely compute the encryptions of ***β***^*†*^ and ***β***_∗_ by pure SIMD additions and multiplications. We note that multiplication of the vectors **B**_*j*_ from the left side and **X**^*T*^**Wz** from the right side with the matrix adj(*A*) can be written as

$$\begin{array}{*{20}l} & {\mathbf{B}}_{j}^{T} \cdot \texttt{adj}(A)\cdot (\mathbf{X}^{T} \mathbf{W} \mathbf{z}) \\ & = \sum_{r,s=1}^{k} b_{jr} \cdot (\texttt{adj}(A))_{r,s} \cdot (\mathbf{X}^{T} \mathbf{W} \mathbf{z})_{s}. \end{array} $$

So, the server evaluates the numerator of Eq. 1 to get the encryption of ***β***^∗^:

11 $$\begin{array}{*{20}l} \texttt{E}(\boldsymbol{\beta}^{*}) \leftarrow & \texttt{E}(|\mathbf{A}|) \cdot \texttt{E}({\mathbf{s}}_{1}^{T} \mathbf{W} \mathbf{z},\ldots,{\mathbf{s}}_{p}^{T} \mathbf{W} \mathbf{z}) -  \\ & \sum_{r,s=1}^{k} \texttt{E}(\mathbf{B}_{r}) \cdot \texttt{E}(\texttt{adj}(\mathbf{A})_{rs}) \cdot \texttt{E}({\mathbf{x}}_{s}^{T} \mathbf{W} \mathbf{z}).  \end{array} $$

Then the output ciphertext E(***β***^∗^) encrypts the values $\boldsymbol {\beta }^{*}_{j}$’s in a way that $\texttt {E}(\boldsymbol {\beta }^{*}) = \texttt {E}(\beta ^{*}_{1}, \beta ^{*}_{2}, \ldots, \beta ^{*}_{p})$. Similarly, we evaluate the denominator of Equation (1) to get an encryption of ***β***^*†*^:

12 $$\begin{array}{*{20}l} \texttt{E}(\boldsymbol{\beta}^{\dagger}) & \leftarrow \texttt{E}(|\mathbf{A}|) \cdot \texttt{E}(c_{1},c_{2},\ldots,c_{p}) \ -  \\ & \quad \sum_{r,s=1}^{k} \texttt{E}(\mathbf{B}_{r}) \cdot \texttt{E}(\texttt{adj}(\mathbf{A})_{rs}) \cdot \texttt{E}(\mathbf{B}_{s}). \end{array} $$

Hence, the output ciphertext E(***β***^*†*^) represents the values $\boldsymbol {\beta }^{\dagger }_{j}$ in a way that $\texttt {E}(\boldsymbol {\beta }^{\dagger }) = \texttt {E}(\beta ^{\dagger }_{1}, \beta ^{\dagger }_{2}, \ldots, \beta ^{\dagger }_{p})$.

#### Output reconstruction

The server sends the resulting ciphertexts E(***β***^∗^),E(***β***^*†*^), and E(|**A**|) to the authority who has the secret key of the underlying HE scheme. Afterwards, the authority decrypts the values and computes the test statistics by using the Wald *z*-test, which are defined by the coefficient estimates divided by the standard errors of the parameters: ${\beta _{j}}/\sqrt {{\texttt {var}_{j}}} = {\beta _{j}^{*}}/\sqrt {|\mathbf {A}| \cdot \beta _{j}^{\dagger }}$ for all *j*∈[*p*]. In the end, the *p*-values can be obtained from the definition $2\cdot \texttt {pnorm}(|{\beta _{j}}|/\sqrt {{\texttt {var}_{j}}})$.

It includes some post computations after decryption, however, we believe that this is a reasonable assumption for the following reasons. Its complexity is even less than that of decryption, so this process does not require any stronger condition on the computing power of the secret key owner. Meanwhile, the output ciphertexts are encrypting (2*p*+1) scalar values, which is two times more information compared to the ideal case. Our solution relies on the heuristic assumption that no sensitive information beyond the desired *p*-values can be extracted from decrypted results. One alternative is that the server can use a masking (sampling random values $r_{j}^{*},r_{j}^{\dagger }, r_{A}$ such that ${r_{j}^{*}}^{2}=r_{j}^{\dagger }\cdot r_{A}$ and multiplying them to $\beta _{j}^{*}, \beta _{j}^{\dagger }$ and |**A**|, respectively) on resulting ciphertexts before sending them to the secret key owner to weaken this assumption.

### Threat model

We consider the following threat models. Firstly, we assume that the computing server is semi-honest (i.e., honest but curious). If we can ensure the semantic security of the underlying HE scheme, there is no information leakage from encrypted data even in malicious setting. Secondly, we assume that the secret key owner does not collude with the server.

## Results

In this section, we explain how to set the parameters and report the performance of our regression algorithms.

### Dataset description

The dataset provided by the iDASH competition organizers consists of 245 samples, partitioned into two groups by the condition of high cholesterol, 137 under control group and 108 under disease group. Each sample contains a binary phenotype along with 10643 SNPs and 3 covariates (age, weight, and height). This data was extracted from Personal Genome Project [25]. The organizers changed the input size in terms of SNPs, cohort size, and threshold of significance to test the scalability of submitted solutions.

We may assume that the imputation and normalization are done in the clear prior to encryption. More precisely, we impute the missing covariate values with the sample mean of the observed covariates. We also center the covariates matrix **X** by subtracting the minimum from each column and dividing by a quantity proportional to the range.

### Parameters settings

We explain how to choose the parameter sets for building secure semi-parallel logistic regression model. We begin with a parameter *L* which determines the largest bitsize of a fresh ciphertext modulus. Since the plaintext space is a vector space of real numbers, we multiply a scale factor of *p* to plaintexts before encryption. It is a common practice to perform the rescaling operation by a factor of *p* on ciphertexts after each (constant) multiplication in order to preserve the precision of the plaintexts. This means that a ciphertext modulus is reduced by log*p* bits after each multiplication or we can say that a multiplication operation consumes one level.

Kim et al. [14] proposed the least squares approach to find a global polynomial approximation of the sigmoid and presented degree 3, 5, and 7 approximation polynomial over the domain [−8,8]. We observed that input values of the sigmoid in our data belong to this interval. As noted in [14], these approximations offer a trade-off between accuracy and efficiency. A low-degree polynomial requires a smaller depth for an evaluation while a high-degree polynomial has a better precision. So, we adapt the degree 3 approximation polynomials of the sigmoid function as *σ*_3_(*x*)=0.5+0.15012*x*−0.001593*x*^3^, which consumes roughly two levels.

Suppose that we start with $\mathbf {v}^{(0)} = {\boldsymbol {\beta }}_{\mathbf {X}}^{(0)}= {\bf {0}} \in \mathbb {R}^{k}$ and the input ciphertext E(**y**^*T*^**X**) is at level *L*. It follows from the parameter analysis of [7] that the ciphertext level of E(***β***_**X**_) after the evaluation of Nesterov’s accelerated GD is *L*−(4·(NUMITER−1)+1) where NUMITER denotes the number of iterations of the GD algorithm. Similarly, we expect each of Steps 1 and 2 to consume two levels for computing the ciphertexts $\texttt {E}(\hat {\mathbf {y}})$ and E(**p**). This means that E(**p**) is at level *L*−(4·NUMITER+1); so, we get

$$\begin{array}{*{20}l} \texttt{lvl}(\texttt{E}(\mathbf{w})) & = L - (4 \cdot \textsc{NumIter} + 2), \\ \texttt{lvl}(\texttt{E}(\mathbf{W}\mathbf{z})) & = L - (4 \cdot \textsc{NumIter} + 3). \end{array} $$

We now consider the replication procedure in Step 3. Although the input vector $\mathbf {w}=(w_{i})_{i=1}^{n}$ is fully packed into a single ciphertext (i.e., the length of the corresponding plaintext vector is *N*_2_), it suffices to produce *n* number of ciphertexts, each of which represents an entry *w*_*i*_ across the entire array. As presented in Section 4.2 of [22], the replication procedure consists of two phases of computation. The first phase is to partition the entries in the input vector into size- 2^*s*^ blocks and construct *n*/2^*s*^ number of vectors consisting of the entries in the *i*-th block with replicated *N*_2_/2^*s*^ times. We use a simple replication operation *n*/2^*s*^ times, which applies multiplicative masking to extract the entry and then perform the AllSum operation to replicate them as in Step 1; its depth is just a single constant multiplication. The second phase is to recursively apply replication operations in a binary tree manner, such that in each stage we double the number of vectors while halving the number of distinct values in each vector; its depth is *s* constant multiplications. In total, we expect to consume (*s*+1) levels during the replication procedure; so, we get

$$\begin{array}{*{20}l} \texttt{lvl}(\texttt{E}(w_{i})) & = L - (4 \cdot \textsc{NumIter} + s + 3), \\ \texttt{lvl}(\texttt{E}(w_{i}z_{i})) & = L - (4 \cdot \textsc{NumIter} + s + 4). \end{array} $$

Later, Step 4 consumes one level from the level lvl(E(*w*_*i*_)) for multiplication; so, we have

13 $$\begin{array}{*{20}l} \texttt{lvl}(\texttt{E}(\mathbf{B}_{\ell})) = L - (4 \cdot \textsc{NumIter} + s + 4).  \end{array} $$

Similarly, Step 5 consumes one more level from the computation of E(*w*_*i*_*z*_*i*_); so we get

$$\texttt{lvl}(\texttt{E}({\mathbf{s}}_{1}^{T} \mathbf{W} \mathbf{z},\ldots,{\mathbf{s}}_{p}^{T} \mathbf{W} \mathbf{z})) = L - (4 \cdot \textsc{NumIter} + s + 5).$$

On the other hand, Step 6 requires one level of multiplication for the evaluation of the update formula (8); so we know

$$\begin{array}{*{20}l} \texttt{lvl}(\texttt{E}(\mathbf{X} \odot \mathbf{W}\mathbf{z})) &= \texttt{lvl}(\texttt{E}(\mathbf{W}\mathbf{z})) - 1 \\ & = L - (4 \cdot \textsc{NumIter} + 4). \end{array} $$

As discussed above, the output ciphertexts $\texttt {E}({\mathbf {x}}_{\ell }^{T} \mathbf {W} \mathbf {z})$ consume (*s*^′^+1) levels during the replication procedure where $\phantom {\dot {i}\!}2^{s'}$ is the unit block size of the first step of the replication procedure; so we have

$$\begin{array}{*{20}l} \texttt{E}({\mathbf{x}}_{\ell}^{T} \mathbf{W} \mathbf{z}) &= \texttt{lvl}(\texttt{E}(\mathbf{X} \odot \mathbf{W}\mathbf{z})) - (s'+1) \\ & = L - (4 \cdot \textsc{NumIter} + s' + 5). \end{array} $$

In Step 7, it requires one and log(*k*−1) levels of multiplications for the evaluation of the update formulas (9) and (10), respectively. If we let *ℓ*^′^=max{lvl(E(**w**)),lvl(E(*Σ*_*r*,*s*,*t*_))}, then we have

$$\begin{array}{*{20}l} \texttt{lvl}(\texttt{E}(\texttt{adj}(\mathbf{A})_{rs})& = {\ell}' - (1 + \log(k-1)), \\ \texttt{lvl}(\texttt{E}(|\mathbf{A}|))& = {\ell}' - (2 + \log(k-1)). \end{array} $$

It follows from the update formulas (11) and (12) in Step 8 that it suffices to set as lvl(E(adj(**A**)_*rs*_))=lvl(E(**B**_*ℓ*_))=3 for obtaining the correct results. This implies that we need to set the number of levels *L* to be at least *L*≥(4·NUMITER+*s*+4)+3 from (13).

In the implementation, we set NUMITER=2,*s*=4,*s*^′^=0, and *L*=19. The encryption levels of data are set as follows:

- lvl(E(**y**^*T*^**X**))=*L*=19,
- lvl(E(**X**))=lvl(E(***β***_**X**_))=14, from (4)
- lvl(E(**y**))=lvl(E(**p**))=10, from (5),
- lvl(E(*x*_*iℓ*_**S**_*i*_))=lvl(E(*w*_*i*_))=4, from (6),
- lvl(E(*Σ*_*r*,*s*,*t*_))=lvl(E(adj(**A**)))+3=6.

We use log*p*_0_≈60, log*q*_0_≈51, and log*q*_*i*_≈43 for *i*=1,…,*L*. Therefore, we derive a lower bound of the bit size of the largest RLWE modulus *Q* as

$$\begin{array}{*{20}l} \log Q & = \log q_{0} + (L-1) \cdot \log q_{i} + \log p_{0} \approx 885. \end{array} $$

Alternatively, we may do a few less or more iterations in the GD algorithm, for example, setting NUMITER=1 or 3. We conducted tests to compare the trade-offs in using different sets of parameters.

We choose the secret key from the ternary distribution, which means to select uniformly at random from {−1,0,1}. The error is sampled from the discrete Gaussian distribution of standard deviation stdev=3.2. We follow the recommended parameters from the standardization workshop paper [26], thus providing at least 128-bits security level of our parameters. We summarize the parameters of our implementation in Table 1. For comparison, we also listed parameters when using NUMITER=1 and 3.

**Table 1:** HE parameter sets

	NumIter	log*N*	*L*	log*p*	log*q*_0_	log*p*_0_	log*Q*
Set-I	1	15	15	43	51	60	713
Set-II	2	15	19	43	51	60	885
Set-III	3	16	23	45	54	62	1106

### Optimization techniques

The standard method of homomorphic multiplication consists of two steps: raw multiplication and key-switching. The first step computes the product of two ciphertexts *ct*(*Y*)=*c*_0_+*c*_1_*Y* and *ct*^′^(*Y*)=*c*0′+*c*1′*Y* (as done in [27]), and returns a quadratic polynomial, called *extended ciphertext*, *ct*_*mult*_=*c*_0_*c*0′+(*c*_0_*c*1′+*c*0′*c*_1_)*Y*+*c*_1_*c*1′*Y*^2^. This ciphertext can be viewed as an encryption of the product of plaintexts with the extended secret (1,*s*,*s*^2^). Afterwards, the key-switching procedure transforms it into a normal (linear) ciphertext encrypting the same message with the secret key (1,*s*).

We observe that the second step is much more expensive than the first one since it includes an evaluation of NTT (Fourier transformation over the modulo space), and that a simple arithmetic (e.g. linear operation) is allowed between extended ciphertexts. To reduce the complexity, we adapt the technique called *lazy key-switching*, which performs some arithmetic over extended ciphertexts instead of running the second step right after each raw multiplication. We get a normal ciphertext by performing only one key-switching operation after evaluating linear circuits over the extended ciphertexts. It can reduce the number of required key-switching algorithms as well as the total computational cost. For instance, if we add many terms after raw multiplications in the right hand side of the update (6) and apply key-switching to the output ciphertext, this takes only one key-switching rather than *n*.

### Performance results

We present our implementation results using the proposed techniques. All the experiments were performed on a Macbook with an Intel Core i7 running with 4 cores rated at 2.5 GHz. Our implementation exploits multiple cores when available, thereby taking the advantages of parallelization.

In Table 2, we evaluated our model’s performance based on the average running time and the memory usages in the key generation, encryption, evaluation, and decryption procedures.

**Table 2:** Experimental results for iDASH dataset with 245 samples, each has 10643 SNPs and 3 covariates (4 cores)

Stage	Set-I		Set-II		Set-III	
Key Generation	4.460 s	2.321 GB	6.665 s	3.584 GB	9.699 s	10.721 GB
Encryption	7.059 s	5.406 GB	7.066 s	6.669 GB	23.023 s	12.137 GB
Training with covariates	2.622 s	7.176 GB	9.367 s	7.186 GB	62.922 s	12.137 GB
Training with all SNPs	40.442 s	10.339 GB	42.567 s	11.176 GB	108.24 s	12.137 GB
**Total evaluation**	**43.064 s**	−	**51.934 s**	−	**171.162 s**	−
Decryption	0.025 s	10.339 GB	0.025 s	11.176 GB	0.055 s	12.137 GB
Reconstruction	0.794 ms	10.339 GB	0.794 ms	11.176 GB	2.821 ms	12.137 GB

**Fig. 1:**
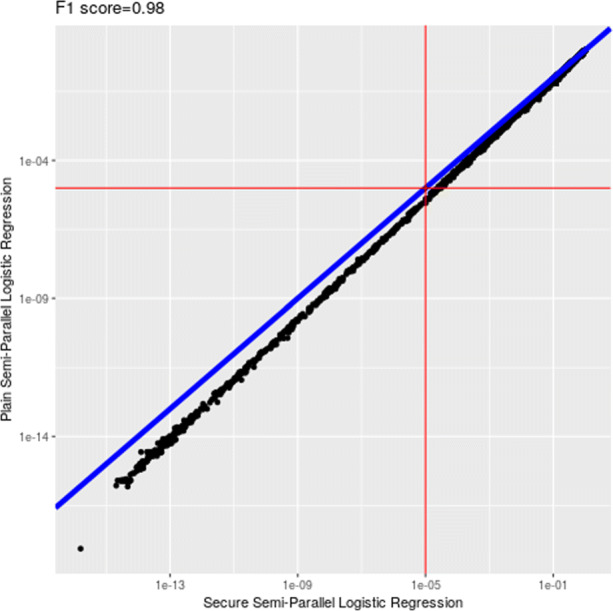
Comparison with the semi-parallel model (*p*-value cut-off: 10^−5^)

**Fig. 2:**
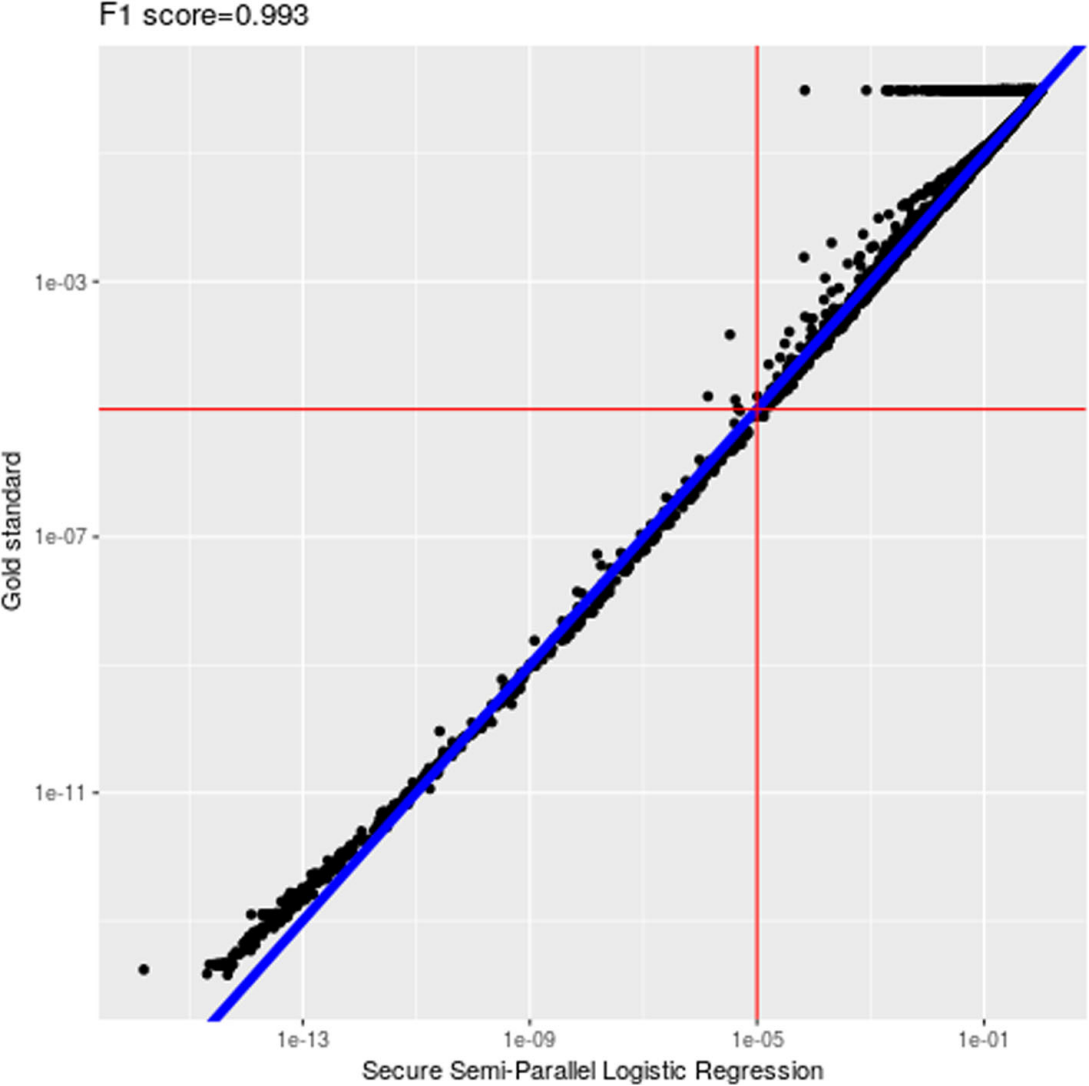
Comparison with the gold standard model (*p*-value cut-off: 10^−5^)

We achieved very high level of accuracy in the final output (after decryption) for all three sets of parameters. The type-I (false positive) and type-II (false negative) errors of the output of our solution are very small when comparing to both the semi-parallel model and the gold standard model (full logistic regression) with respect to various *p*-value cut-off thresholds. See Figs. 1 and 2 for comparisons against these two plain models with a cut-off of 10^−5^ when NUMITER=2. To better compare the estimated *p*-values (above or below certain cut-offs) on the encrypted model against the plaintext one (semi-parallel GWAS), we measured *F*_1_-scores on the *p*-values obtained from our solution against the two plain models. The resulting *F*_1_-scores are very close to 1 across all cases with different cut-offs (10^−2^ to 10^−5^), which are shown in Table 3.

**Table 3:** *F*_1_-Scores on different models

Cut-off	v.s. Plain semi-parallel model			v.s. Plain gold standard model		
	Set-I	Set-II	Set-III	Set-I	Set-II	Set-III
10^−2^	0.9807	0.9830	0.9964	0.9818	0.9808	0.9710
10^−3^	0.9749	0.9810	0.9975	0.9878	0.9887	0.9740
10^−4^	0.9745	0.9798	0.9969	0.9878	0.9888	0.9729
10^−5^	0.9828	0.9852	0.9971	0.9946	0.9970	0.9805

We also conducted the DeLong’s test [28, 29] to validate our solution against the semi-parallel model. Specifically, we drawn at uniformly random about 10% of the total SNP test data and transformed the corresponding *p*-values to 0-1 labels according to the cut-off threshold; then we constructed the ROC (Receiver Operating Characteristic) curves for these labels and performed the DeLong’s test to compare the AUCs (Area Under the Curve) of these curves. Such test was repeated 10 times to obtain the mean and the standard deviation of the *p*-values of the test. The results for NUMITER=2 are shown in Table 4.

**Table 4:** DeLong’s Test for AUCs of our solution with Set-II against the plain semi-parallel model

Cut-off	Mean and stdev of the test results
10^−2^	0.4038 ±0.3001
10^−3^	0.5357 ±0.2704
10^−4^	0.6404 ±0.2638
10^−5^	0.8959 ±0.2195

## Discussion

One constraint in our approach is that the matrix inverse can be computed in an efficient way when the input dimension is small. In modern GWAS, it is common to include covariates to account for such factors as gender, age, other clinical variables and population structure. A significant challenge in performing efficient secure GWAS on this generalized model is to handle large-scale matrix inversion.

## Conclusion

In this paper, we showed the state-of-the-art performance of secure logistic regression model training for GWAS. We have demonstrated the feasibility and scalability of our model in speed and memory consumption. We expect that the performance can be improved if the underlying HE scheme is rewritten with optimized code.

## Data Availability

Not applicable.
